# Gastrodin protects against LPS-induced acute lung injury by activating Nrf2 signaling pathway

**DOI:** 10.18632/oncotarget.16740

**Published:** 2017-03-31

**Authors:** Zhuo Zhang, Jie Zhou, Daqiang Song, Yuhong Sun, Changli Liao, Xian Jiang

**Affiliations:** ^1^ Laboratory of Pharmacology, College of Pharmacy, Southwest Medical University, Luzhou, Sichuan, China; ^2^ Laboratory of Science of Chinese Pharmacology, College of Pharmacy, Southwest Medical University, Luzhou, Sichuan, China; ^3^ Research Department, College of Pharmacy, Southwest Medical University, Luzhou, Sichuan, China; ^4^ Department of Anesthesiology, The Affiliated Hospital of College of Pharmacy, Southwest Medical University, Luzhou, Sichuan, China

**Keywords:** gastrodin, LPS, Nrf2, lung injury

## Abstract

Gastrodin (GAS), a phenolic glucoside derived from *Gastrodiaelata Blume*, has been reported to have anti-inflammatory effect. The aim of this study was to investigate the effects of GAS on LPS-induced acute lung injury in mice. ALI was induced by the intranasal administration of LPS and GAS was given 1 h or 12 h after LPS treatment. The results indicated that GAS treatment markedly attenuated the damage of lung injury induced by LPS. GAS attenuated the activity of myeloperoxidase (MPO) and down-regulated the levels of pro-inflammatory cytokines TNF-α, IL-6 and IL-1β in BALF. LPS-induced lung edema and lung function were also reversed by GAS. Furthermore, GAS was found to inhibit LPS-induced inflammatory cells infiltration. In addition, treatment of GAS inhibited LPS-induced NF-κB activation and up-regulated the expression of Nrf2 and HO-1. In conclusion, our results indicated that GAS had anti-inflammatory effects on LPS-induced acute lung injury. The anti-inflammatory mechanism of GAS was through the inhibition of NF-κB and activation of Nrf2 signaling pathways.

## INTRODUCTION

Acute lung injury (ALI), the inflammation of the lung tissue, is a highly prevalent disease [1]. It is characterized by hypoxemia and pulmonary edema in lung [2]. This leads to a significant cause of mortality in critically ill patients. Recent studies showed that inflammatory response played a critical role in the pathogenesis of ALI [3, 4]. LPS induces the release of inflammatory cytokine including TNF-α and IL-1β, which exacerbate harmful immune responses [5, 6]. Previous studies suggested that inhibition the release of inflammatory cytokines could treat ALI [5, 7]. The production of inflammatory cytokines was regulated by NF-κB activation [8]. Inhibition of NF-κB activation could attenuate LPS-induced ALI [9].

Nrf2 is a nuclear factor that has been reported to control the expression of detoxifying enzymes [10]. Activation of Nrf2 could up-regulate the expression HO-1 [11]. Activation of Nrf2 signaling pathway could inhibit NF-κB activation and inflammatory response [12]. Many herbal compounds protected against LPS-induced ALI by activation Nrf2 signaling pathway [13, 14]. Therefore, Nrf2 may be a promising target for treating lung injury. Recent studies showed that ALI was a kind of medium disease with mortality as high as 35~40% despite optimal supportive care, which caused great threat to people's lives and health [15, 16]. Therefore, it is urgently to search for the safe and effective agent for the intervention of ALI.

In recent years, herbal drugs have become increasingly popular and their use is widespread [17]. A number of herbals and its natural compound have been reported to have anti-inflammatory effects [18, 19]. Furthermore, many herbal medicine have the ability to attenuate LPS-induced ALI [20]. Gastrodin (GAS), a phenolic glucoside derived from *Gastrodiaelata Blume*, has been reported to have anti-inflammatory, anti-nociceptive, and anti-oxidative effects [21, 22]. Previous study showed that GAS attenuated the inflammatory response in H9c2 cardiomyocytes [23]. Also, GAS was found to improve anti-oxidant and anti-inflammation activities and inhibit apoptosis pathway in cerebral ischemic damage [22]. Furthermore, it has been reported that GAS protects against MPP+-induced oxidative stress in human dopaminergic cells [24]. GAS inhibits expression of inflammatory cytokines in LPS-stimulated microglia [25]. However, there have been no reports on the effects of GAS on LPS-induced acute lung injury. In this study we investigated the protective effects of GAS on LPS-induced ALI in mice.

## RESULTS

### The effects of gastrodin on histopathological changes

**Figure 1:**
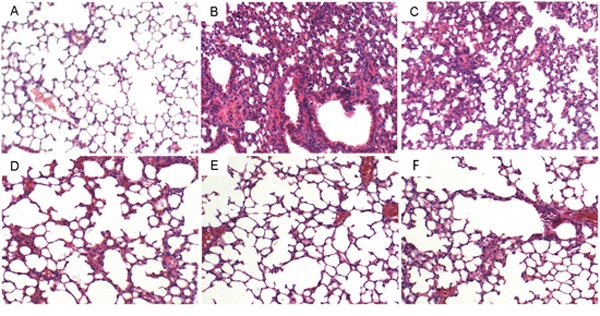
Effects of gastrodin on histopathological changes in lung tissues in LPS-induced ALI mice Representative histological changes of lung obtained from mice of different groups. **(A)** Control group, **(B)** LPS group, **(C)** LPS+ gastrodin (15 mg/kg) group, **(D)** LPS + gastrodin (30 mg/kg) group **(E)** LPS + gastrodin (60 mg/kg) group **(F)** LPS+gastrodin (60mg/kg, 12 h later) (Hematoxylin and eosin staining, magnification 200×).

Lung tissues were collected at 12 h or 24 h after LPS treatment. Then the lung tissues were subjected to H&E staining. Compared to the control group, LPS group showed severe histopathologic changes, including thicken of the alveolar wall, pulmonary congestion and alveolar wall thickness (Figure 1B). However, treatment with gastrodin (15, 30 and 60 mg/kg) significantly ameliorated LPS-induced histopathological changes (Figure 1C, 1D, 1E). Treatment with gastrodin (60 mg/kg) 12 h after LPS treatment also significantly ameliorated LPS-induced histopathological changes (Figure 1F).

### The effects of gastrodin on MPO activity and lung wet to dry weight ratio

**Figure 2:**
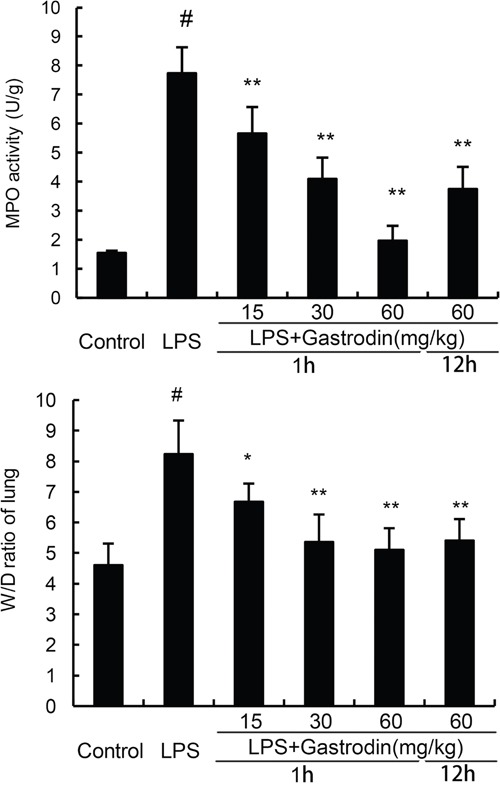
Effects of gastrodin on MPO activity in lung tissues and lung wet to dry weight ratio of LPS-induced ALI The values presented are the mean ± SEM (n=12 in each group) of three independent experiments. p#<0.01 vs. control group, p**<0.01 vs. LPS group.

MPO, a functional biomarker of neutrophils, was tested to assess the neutrophil accumulation in lung. We found that the MPO activity was significantly up-regulated in the LPS group compared with the control group. However, gastrodin treatment significant inhibited the activation of MPO (Figure 2A). We also detected the effects of gastrodin on lung wet to dry weight ratio. The results showed that lung wet to dry weight ratio was significantly up-regulated in the LPS group compared with the control group. However, gastrodin treatment significant inhibited LPS-induced lung wet to dry weight ratio (Figure 2B).

### Effects of gastrodin on lung function

**Figure 3:**
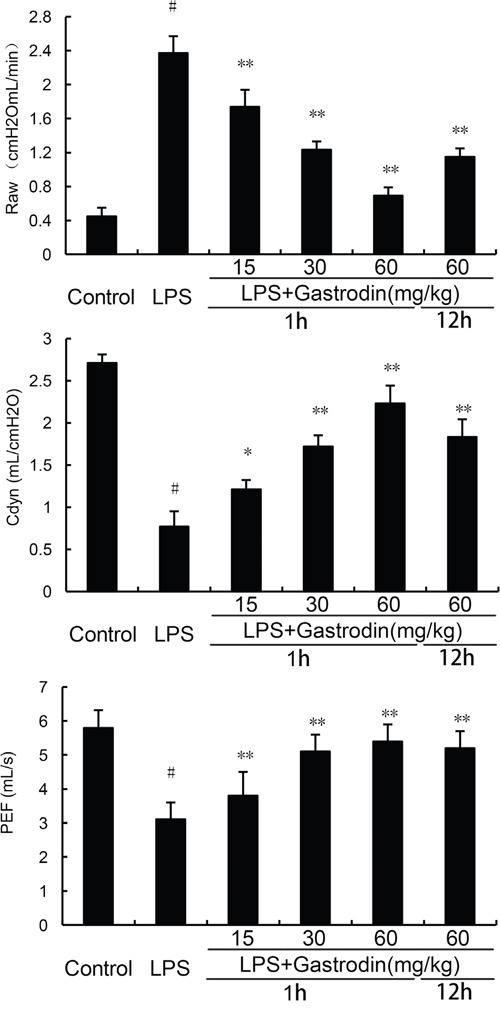
Effects of gastrodin on lung function The values presented are the means ± SEM of three independent experiments. p#<0.01 vs. control group, p**<0.01 vs. LPS group.

The effects of gastrodin on lung function were detected in this study. As shown in Figure 3, LPS significantly increased Raw and treatment of gastrodin inhibited LPS-induced Raw. Cdyn and PEF were decreased by LPS. Treatment of gastrodin significantly reversed the LPS-induced decrease of Cdyn and PEF.

### Effects of gastrodin on inflammatory cell count in the BALF

**Figure 4:**
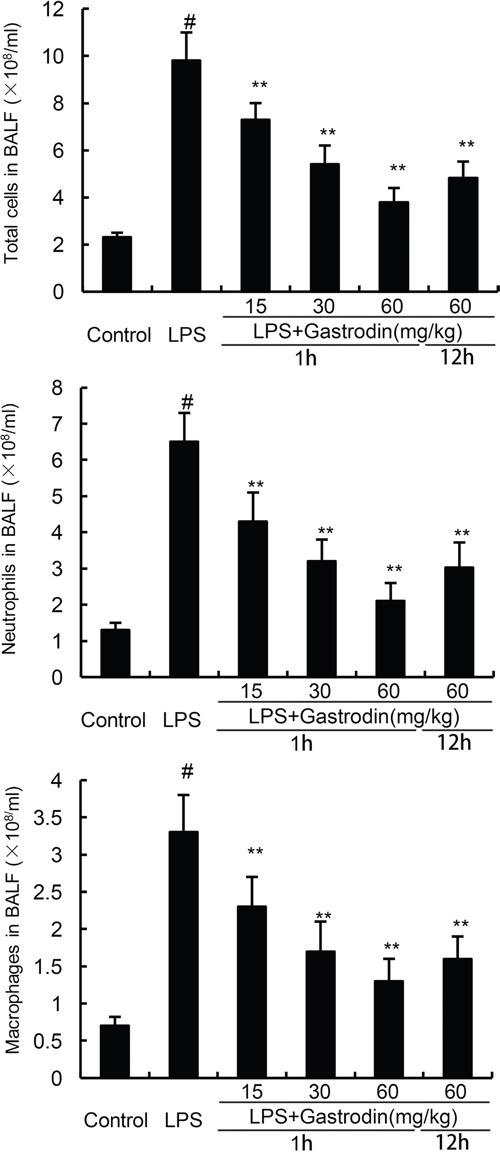
Effects of gastrodin on inflammatory cell count in the BALF of LPS-induced ALI mice The values presented are mean ± SEM (n=12 in each group) of three independent experiments. p#<0.01 vs. control group, p*<0.05, p**<0.01 vs. LPS group.

Effects of gastrodin on inflammatory cell count in the BALF were measured in this study. As shown in Figure 4, the numbers of total cells, neutrophils, and macrophages increased significantly after LPS treatment. However, treatment of gastrodin significantly inhibited LPS-induced inflammatory cells infiltration in BALF (Figure 4).

### Gastrodin inhibits inflammatory cytokines release in BALF

**Figure 5:**
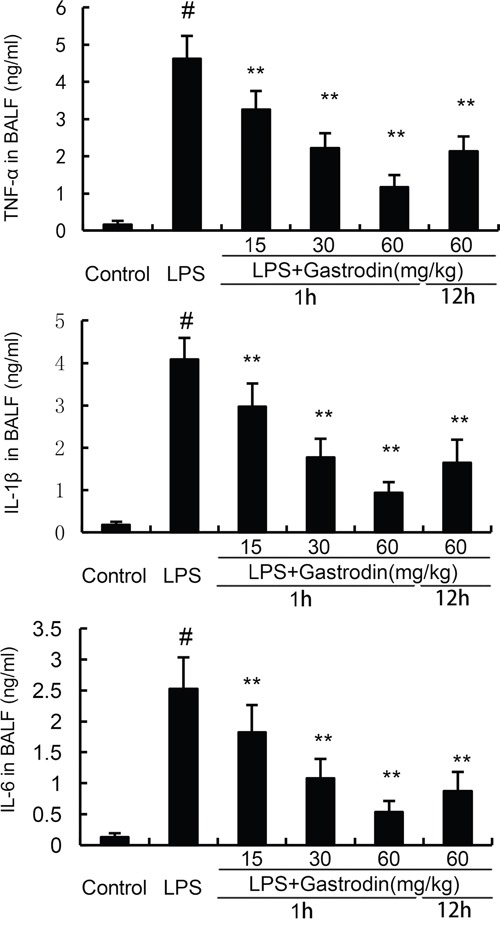
Effects of gastrodin on TNF-α, IL-1ß, and IL-6 production in the BALF of LPS-induced ALI mice The values presented are mean ± SEM (n=12 in each group) of three independent experiments. p#<0.01 vs. control group, p**<0.01 vs. LPS group.

Inflammatory cytokines has been reported to play critical roles in ALI. In the present study, the levels of TNF-α, IL-6, and IL-1β in BALF were measured by ELISA assays. Compared with the control group, the levels of TNF-α, IL-6, and IL-1β in BALF were increased in the LPS group. However, gastrodin treatment significantly inhibited the levels of inflammatory cytokines TNF-α, IL-6, and IL-1β (Figure 5).

### Gastrodin inhibits NF-κB activation in LPS-induced ALI

**Figure 6:**
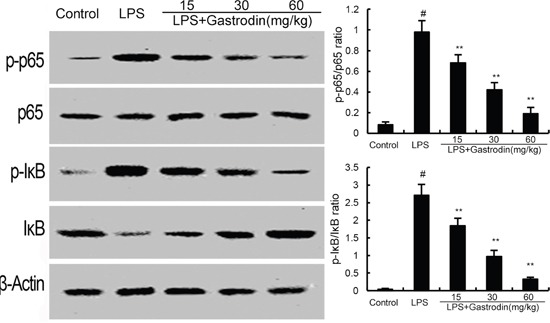
Gastrodin inhibits LPS-induced NF-κB activation The values presented are the means ± SEM of three independent experiments. p#<0.01 vs. control group, p**<0.01 vs. LPS group.

To investigate the anti-inflammatory mechanism of gastrodin, western blot analysis was applied to investigate the effects of gastrodin on LPS-induced NF-κB activation in lung tissues. The results showed thatLPS obviously increased the phosphorylation of NF-κBp65 and I-κBα. However, gastrodin treatment significantly inhibited the phosphorylation of NF-κBp65 and I-κBα (Figure 6).

### The effects of gastrodin on Nrf2 and HO-1 expression

**Figure 7:**
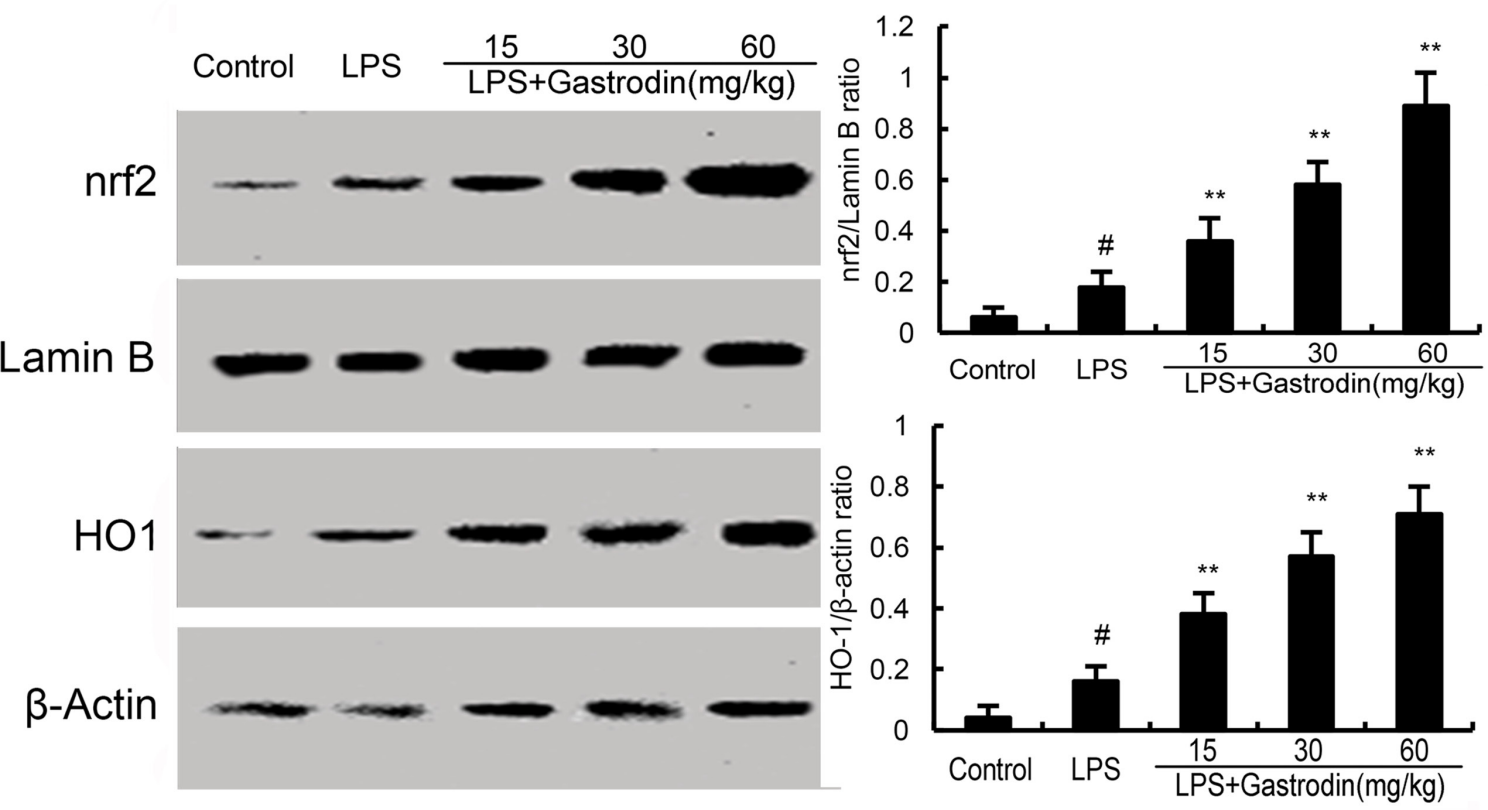
Effects of gastrodin on Nrf2 signaling pathway The values presented are the means ± SEM of three independent experiments. p#<0.01 vs. control group, p**<0.01 vs. LPS group.

The effects of gastrodin on Nrf2 signaling pathway were measured by western blot analysis. As shown in Figure 5, compared with the control group, the expression of Nrf2 and HO-1 increased in LPS-treated group. However, gastrodin dose-dependently increased the expression of Nrf2 and HO-1 (Figure 7).

## DISCUSSION

Acute lung injury (ALI) is a major clinical problem in the intensive care unit [26]. The mortality of ALI remains high in recent years. A large body of evidences showed that inhibition of inflammation may have protective effects against ALI [27]. In this study, we demonstrated that gastrodin protected against LPS-induced ALI by inhibiting inflammatory response.

LPS challenge leads to lung edema and inflammatory cells infiltration [28]. The infiltration of neutrophils results in the releasing of reactive oxygen species and inflammatory cytokines, which lead to lung injury. MPO was used as a marker for tissue neutrophils content [29]. Previous study showed that inhibition of neutrophils infiltration could attenuate LPS-induced lung injury [30]. In the present study, our results showed that gastrodin significantly inhibited LPS-induced inflammatory cells infiltration and MPO activity. Inflammatory cytokines are known to be involved in the development of ALI [31]. In the model of LPS-induced ALI, inflammatory cytokines in BALF increased significantly [32]. These inflammatory cytokines TNF-α, IL-1β, and IL-6 are associated with lung injury severity [33]. In this study, our results showed that gastrodin significantly inhibited LPS-induced inflammatory cytokines production. Furthermore, LPS-induced lung histopathological changes were attenuated by treatment of gastrodin. These results suggested that gastrodin had protective effects against LPS-induced ALI.

NF-κB, an essential transcription factor, is a critical molecule in the regulation of inflammation [34, 35]. In normal conditions, NF-κB remains in the cytoplasm with IκBα. Once stimulated by LPS, it will result in the release of NF-κB through phosphorylation and degradation of IκBα [36]. Accumulating studies have provided strong evidence that NF-κB was involved in the development of ALI [37]. Previous study showed that inhibition of NF-κB activation could attenuate the LPS-induced ALI [28]. To investigate the anti-inflammatory mechanism of gastrodin, the effects of gastrodin on NF-κB were measured. In the present study, we demonstrated that gastrodin significantly inhibited LPS-induced NF-κB activation. The results suggested that gastrodin inhibited LPS-induced inflammatory cytokines production via inhibiting NF-κB signaling pathway.

Nrf2 signaling pathway is a promising approach to protect against ALI, which regulates the oxidative and inflammatory responses [38, 39]. Activating of Nrf2 could regulate the expression of antioxidant and anti-inflammatory genes. Studies showed that many herbal compounds protected against LPS-induced lung injury by activating Nrf2 signaling pathway [40, 41]. Furthermore, it has been shown that Nrf2 has the ability to regulate the activation of NF-κB [42]. It has been reported that transfection with HO-1 siRNA lead to the inhibition of NF-κB activation [43]. To further investigate the anti-inflammatory mechanism of gastrodin, Nrf2 signaling pathway was detected in this study. We found that gastrodin dose-dependently increased the expression of Nrf2 and HO-1. These results demonstrated that gastrodin attenuated LPS-induced ALI by activating Nrf2 signaling pathway.

In conclusion, gastrodin treatment attenuates LPS-induced ALI in mice by inhibiting inflammatory cytokines production. The anti-inflammatory mechanism of gastrodin is through activating Nrf2, which subsequently inhibits LPS-induced NF-κB activation. Gastrodin might be an effective agent for the treatment of ALI.

## MATERIALS AND METHODS

### Reagents

Gastrodin (purity>98%) was purchased from Sigma-Aldrich (St. Louis, MO, USA). LPS was purchased from Sigma Chemical Co. (St. Louis, MO, USA). Antibodies for Nrf2, HO-1, NF-κB p65, IκBα, and β-actin were purchased from Santa Cruz Biotechnology (Autogen, Bioclear, UK). Mouse TNF-α, IL-6 and IL-1β ELISA kits were obtained from R&D (R&D systems, Minneapolis, MN, USA). MPO detection kit was obtained from Nanjing Jiancheng Bioengineering institute (Nanjing, China).

### Acute lung injury model

BALB/c mice, 6-8 weeks old, were purchased from Guangzhou Medical University (Guangzhou, China). The mice were given a standard diet and received water free access. All animal experiments were performed according to the Committee of Animal Experimentation of the Southwest medical university. Seventy-two mice were randomly divided into six groups and each group contains twelve mice: control group, LPS group, LPS + gastrodin (15, 30 and 60 mg/kg) groups, LPS + gastrodin (60 mg/kg) group (12 h later). 10 μg of LPS was instilled intranasally (i.n.) in 50 μl PBS to induce lung injury. Gastrodin (15, 30 and 60 mg/kg) was given 1 h or 12 after LPS treatment. Then, the mice were euthanized and the lung tissues and BALF were collected for the subsequent experiments.

### Histopathologic evaluation of lung tissues

To detect histopathological changes, lung tissues were collected and fixed in 10%formalin for 12 h, imbedded in paraffin and sliced. The sections were stained with hematoxylin and eosin (H&E) staining. Finally, pathological changes in the lung tissues were examined under a light microscope.

### MPO assay

12h after LPS challenge, the lung tissue samples were collected, for measuring MPO activity. MPO activity, represents the parenchymal infiltration of neutrophils, were examined by using the MPO determination kit (Nanjing Jiancheng Bioengineering institute, China) following the manufacturer's protocols.

### Lung wet-to-dry weight ratio

The lung tissues of mice were excised, blotted dry, weighed to obtain the ‘wet’ weight. Then, the lung tissues were placed in an oven at 80°C for 72 h to obtain the ‘dry’ weight. The ratio of wet lung to dry lung was calculated to assess lung edema.

### Collection of BALF and cell counting

The BALF were obtained by intratracheal instillation with 0.8 ml cold PBS. Then the BALF samples were centrifuged and the supernatants were used to analysis the levels of cytokines. The cell pellets were suspended with PBS for cell counts. Neutrophils and macrophages in BALF were stained with the Kwik-Diff staining set (Thermo).

### Lung function assay

Lung function including air resistance (Raw), lung dynamic compliance (Cdyn), and peak expiratory flow (PEF) was detected using small animal spirometer (PLY3211 system, Buxco Electronics, USA) as previously described [44].

### Inflammatory cytokines assay

The BALF were harvested 12 h after LPS treatment and the levels of TNF-α, IL-1ß, and IL-6 in the BALF were measured by ELISA kits in accordance with the manufacturer's instructions (BioLegend, CA, USA).

### Western blot analysis

The total proteins were extracted from lungs using Protein Extraction Kit according to the manufacturer's protocol (Beyotime, Haimen, China). Protein concentrations were measured using the BCA protein assay kit. The protein samples with equal amounts (40 μg) of protein were separated on 12% SDS gel and transferred onto PVDF membranes. After blocking with 3% nonfat milk in TBST for 2 h at room temperature, the membrane was probed with the primary antibodies and HRP-conjugated secondary antibodies. The signals on the membranes were developed with the ECL Plus detection reagents (Thermo).

### Statistical analyses

Comparison between groups were analyzed using one-way analysis of variance (ANOVA) and the two-tailed Student's t test. All data are presented as mean ± SEM, with a p value <0.05 considered to be statistically significant.
